# Sm16, a major component of *Schistosoma mansoni* cercarial excretory/secretory products, prevents macrophage classical activation and delays antigen processing

**DOI:** 10.1186/s13071-014-0608-1

**Published:** 2015-01-06

**Authors:** David E Sanin, Adrian P Mountford

**Affiliations:** Centre for Immunology and Infection, Department of Biology, University of York, York, UK

**Keywords:** Helminths, Macrophages, Toll like receptors, Schistosoma mansoni, Cercarial excretory/secretory products, Macrophage activation

## Abstract

**Background:**

*Schistosoma mansoni* cercariae penetrate the skin by releasing excretory/secretory (E/S) products known as 0-3hRP, which are associated with immune modulation through Toll like receptor (TLR) signalling. Furthermore, these secretions contain Sm16, which when given to cells as a recombinant protein inhibits human monocyte derived cytokine responses to TLR4 and TLR3 ligands. Nonetheless, the extent and mechanism(s) of these inhibitory effects remain largely uncharacterized.

**Methods:**

Murine bone marrow derived macrophages were exposed to different fractions of 0-3hRP, obtained via ultracentrifugation, or recombinant Sm16. These cells were exposed to the parasite molecules in combination with different TLR ligands, or Interferon gamma, and tested for the production of the cytokines IL-10 and IL-12p40, and their ability to process antigen.

**Results:**

The immunomodulatory function of 0-3hRP is enriched predominantly in the pellet fraction, which contains a greater proportion of Sm16, also corroborating the ability of recombinant Sm16 to inhibit macrophage activation in response to TLR ligands. We further demonstrate that Sm16 blocks classical activation of macrophages to LPS or IFN-γ stimulation *in vitro,* and that inhibition of macrophage classical activation is independent of TLR2 recognition. Finally we show that Sm16 shares the altered intracellular processing observed for 0-3hRP, and is able to delay antigen processing by macrophages.

**Conclusions:**

Collectively, our findings show that Sm16 is a major component of *S. mansoni* cercarial E/S products, and is partly responsible for its immune-regulatory properties. Moreover, we propose that the mechanism employed by Sm16 to exert its inhibitory function is likely to be linked with alteration of endosomal trafficking and is not dependent on particular TLR receptors. Finally, we suggest that accumulation of Sm16 in the skin after percutaneous infection with *S. mansoni* cercariae could contribute to limiting dermal inflammation.

**Electronic supplementary material:**

The online version of this article (doi:10.1186/s13071-014-0608-1) contains supplementary material, which is available to authorized users.

## Background

*Schistosoma mansoni* cercariae penetrate the skin by releasing excretory/secretory (E/S) products derived from the post and pre-acetabular glands of the parasite [1–3] which aid migration of larvae through the skin to reach blood vessels, thereby facilitating infection of the host [4,5]. *S. mansoni* cercarial E/S products contain more than 50 different proteins [1,6], and are largely released within the first three hours after transformation, hence this preparation has been termed 0–3 hour released preparation (0-3hRP) [7]. These molecules are the first parasite-derived material encountered by innate immune cells (e.g. macrophages, neutrophils, and dendritic cells) in the skin and as such constitute the first line of defense against invading parasites. For example, macrophages in the skin take up secreted *S. mansoni* E/S products [3]. These secretions also induce a strong cytokine response from macrophages in a Toll like receptor (TLR) dependent manner [7] and are retained in early endosomes for longer than other immunogenic stimuli [3] suggesting that 0-3hRP conditions the way immune cells affect their environment and process phagocytized material [8].

The constituent(s) of 0-3hRP responsible for its potential immunomodulatory function are not known but several constituent molecules have theoretical roles [1,2]. Enzymes which allow remodeling of extracellular matrix necessary for parasite penetration of the skin are some of the most frequently identified proteins, of which the best studied is cercarial elastase with chymotrypsin activity enabling it to break skin elastin [5]. However, at least seven other elastases are secreted by larvae into the skin, alongside five metalloproteases, one of which is Invadolysin [2]. The only protein in 0-3hRP with a defined immunological function is Sm16 (Smp_113760), which is able to induce cell apoptosis if it reaches the cytosol [9], and effectively blocks signalling downstream of TLR4 and TLR3 in human monocytic cell lines [10]. Sm16 is expressed between the late developmental stage of the sporocyst in the intermediate molluscan host and the invading larvae, disappearing after 48 h of skin penetration [11]. Sm16 interference with cytokine responses by human monocytes is upstream of IRAK1 activation and NF-κB signalling [10], indicating that it’s functions occur in close association with the earliest events of TLR signal transduction.

0-3hRP is recognized by TLRs, whilst Sm16 is known to inhibit the function of these receptors [7,10]. Consequently, we investigated the distribution and function of Sm16 in different fractions of *S. mansoni* cercarial E/S products. The immunomodulatory function of 0-3hRP is enriched predominantly in its pellet fraction and we show that this fraction retains a greater proportion of Sm16 than the soluble fraction. We corroborate the ability of this protein to inhibit macrophage activation in response to TLR ligands, and further demonstrate that Sm16 is able to block classical activation of macrophages *in vitro* and that it functions independently of TLR2 recognition. Finally, we determine that Sm16 shares the altered intracellular processing as seen with 0-3hRP, and has the potential to delay antigen processing. Collectively, our findings show that Sm16 is a major component of *S. mansoni* cercarial E/S products, with this protein being partly responsible for the regulatory function of these secretions.

## Methods

### Parasites and parasite-derived material

The life cycle of a Puerto Rican strain of *Schistosoma mansoni* (*S. mansoni*) was maintained in outbred NMR-I mice and *Biomphalariaglabrata* snails. Infective cercariae were obtained following exposure of snails with a patent infection to incandescent light for 2 h to induce the release of the parasites. Cercarial E/S products were produced as described previously [1,3,7]. Briefly, culture supernatants containing the 0–3 hour released preparation (0-3hRP*)* were collected (ensuring whole larvae and parasite tails were discarded), and stored at −20°C until required. Pooled supernatants were concentrated using filter spin columns with a molecular weight cut off of 3 kDa (GE Life Sciences) and the protein content measured using the BCA® protein assay (Thermo Scientific).

Recombinant Sm16 (rSm16), unlabelled or labelled with AlexaFluor® 546, was a gift from Dr Martin Gullberg, Umeå University, Sweden [9,10].

### Fractionation of 0-3hRP

0-3hRP was fractionated by centrifugation at 100,000 *g* for 1 hour at 4°C into a soluble preparation and a pellet. The soluble 0-3hRP preparation was denoted 0-3hRP_S_, whilst the pellet re-suspended using a vortex in an equivalent volume of PBS was denoted 0-3hRP_P_. The protein content of both preparations was quantified as specified above.

### SDS polyacrylamide gel electrophoresis (PAGE)

0-3hRP and its fractions were separated by SDS-PAGE under reducing conditions (1x NuPAGE® Sample Reducing Agent; Life Technologies) on 4-12% NuPAGE® Bis-Tris Precast gels (Life Technologies) for 2 h at 200 V in 1xNuPAGE® MOPS SDS Running Buffer (Life Technologies). Gels were stained over-night using Brilliant Blue G concentrate (SIGMA), and imaged using a GelDoc® and ImageLab® by Biorad.

Selected protein bands were identified by tandem mass spectrometry (MS/MS) by the Proteomics division of the Bioscience Technology Facility (University of York, York, UK) using a Matrix assisted laser desorption ionization (MALDI)-MS and MS/MS are performed using a Bruker ultraflex III MALDI-Time of flight (TOF)/TOF.

### Western blot analysis

0-3hRP fractions and rSm16 were transferred after SDS-PAGE onto nitrocellulose membranes using an iBlot® Transfer Stack (Life Technologies). The membranes were then processed using the SnapID® system (Millipore) blocked with PBS containing 1% BSA, incubated first with rabbit anti-rSm16 antibody (1:5000) (gift from Dr Martin Gullberg, Umeå University, Sweden) for 10 min, and then goat anti rabbit antibody (1:30000) conjugated to horseradish peroxidase (Abcam). SuperSignal® West Pico chemiluminescence reagent (Thermo Scientific) was used to reveal labelled proteins using X-ray film imaging (GE Healthcare).

### Animals

All animals were bred and maintained in the Biological Services Facility at the Department of Biology, University of York, according to the standards laid out in the Animal’s Scientific Procedures Act 1986, and housed in filter-topped cages under specific pathogen free conditions. The University of York Ethics committee approved all experimental work. Aged matched female C57BL/6 strain, or TLR2 deficient (TLR2^−/−^) [12] mice between 6–10 weeks old were used for all experimental procedures.

### *In vitro* culture and stimulation of murine bone marrow-derived macrophages

Bone marrow from both femurs and tibias was flushed with PBS using a 25G needle and the resulting cell suspensions filtered to remove bone and tissue debris. Aliquots of 5×10^6^ cells were re-suspended in DMEM® medium (Gibco) containing 10% FCS, 2 mM L-glutamine (Gibco), 1% Pen/Strep (Gibco) and 50 μM 2-mercaptoethanol (complete DMEM), supplemented with macrophage colony stimulating factor (M-CSF) obtained from culture supernatants of L929 murine fibroblast cell line. Bone marrow cell suspensions were cultured at 37°C and 5%CO_2_ in 10 cm culture dishes for 7 days prior to the collection of adherent cells which were re-suspended in complete DMEM and subsequently used as bone marrow derived macrophages (BMMΦs). Stimulation assays were performed on 1×10^5^ BMMϕs/well (96 well plate) in 200 μl of complete DMEM, containing different concentrations of parasite-derived material, or recombinant Sm16 (rSm16). BMMϕs were stimulated in the same manner with 1 ng/ml lipopolysaccharide (LPS) (SIGMA-ALDRICH, from *Escherichia coli* 0111:B4), 25 μg/ml Polyinosinic:polycytidylic acid (Poly I:C) (SIGMA-ALDRICH) and 5 μg/ml Pam3CSK4 (InvivoGen).

### Flow cytometry

BMMϕs were incubated in round bottom 96 well plates with neat goat serum and 1 μg anti CD16/CD32 monoclonal antibody (mAb) (eBiosciense), for 10 min at 4°C to prevent non-specific mAb binding to Fc-receptors. Cells were then labelled with mAb against F4/80 (BM8), CD11b (M1/70), CD11c (HL3) and MHC-II (M5/114) (all eBiosciences) in 10 μl of 1% FCS in PBS (FACS buffer) for 30-45 min at 4°C. Cells were washed and then subject to immediate acquisition by flow cytometry, or fixed in 100 μl 2% paraformaldehyde (PFA) in PBS to enable acquisition at a later point. Antigen processing assays were carried out by exposing rSm16 treated or control BMMϕs to 100 AlexaFluor®488 conjugated *E. coli* BioParticles® (Life technologies) per cell for varying lengths of time. Cells were then fixed as described above and analysed by flow cytometry. All flow cytometry was acquired using the Cyan ADP analyser (DakoCytomation, Stockport, UK). Data was analysed using FlowJo software v7.6.5 (Tree Star, Inc, Ashland, Oregon, USA).

### Enzyme linked immune absorbent assays

Culture supernatants were collected from *in vitro* BMMϕs cultures after 24 hours, as described above, for cytokine analysis. The amounts of IL-10 and IL-12p40 were determined using DuoSet ELISA kits (R&D Systems).

### Griess assay

The amount of nitrite as an indirect product of the production of NO, was measured using Griess Reagent kit (Life Technologies). Briefly, culture supernatants were incubated for 30 min with Griess reagent, alongside a standard curve supplied by the manufacturer. Absorbance was measured at 550 nm, and concentrations estimated based on the standard curve.

### Confocal microscopy of BMMΦs exposed to fluorescently labelled rSm16

BMMΦs were allowed to adhere to glass cover slips for two hours in 24 well plates (1×10^6^ cells/well) and then exposed to labelled rSm16 (50 μg/ml) and/or Fluorescein isothiocyanate (FITC) conjugated DEXTRAN (DEXTRAN^FITC^) (SIGMA) for different periods of time. After washing, cells were fixed on to the cover slips for 20 min with 4% PFA in PBS at room temperature. Cells were then incubated with DAPI (2 μg/ml) (SIGMA) for 5 min, mounted onto a glass microscope slide using Prolong® Gold (Life technologies), sealed with nail varnish, and finally imaged.

Alternatively, cover slips were placed in 0.05% saponin 0.2% BSA (staining buffer) for 30 min at room temperature and then incubated for 1 hour with polyclonal rabbit antibody against Early endosome antigen (EEA)-1 (Abcam) (1:200). Cover slips were washed 3x and then probed for 1 hour with goat anti-rabbit Alexa Fluor® 488 (Life technologies) (1:1000). Finally, cover slips were washed twice with DAPI (2 μg/ml) included in the second washing step. After rinsing with deionized water, cover slips were mounted on glass slides using Prolong® Gold as above.

All images were acquired using a Zeiss LSM 710 invert microscope using ZEN microscope software.

### Statistical analysis

Analysis of Variance (ANOVA) and multiple comparisons tests (Two tailed T-test, Tukey’s, Sidak’s and Bonferroni’s) were performed to establish statistically significant differences between the groups (* = p < 0.05, ** = p < 0.01; *** = p < 0.001, **** = p < 0.0001) using the software package GraphPad Prism®. Error bars represent the standard error of the mean (SEM), based on technical replicates.

## Results

### Pellet fraction of *S. mansoni* E/S products induces IL-10 production

**Figure 1:**
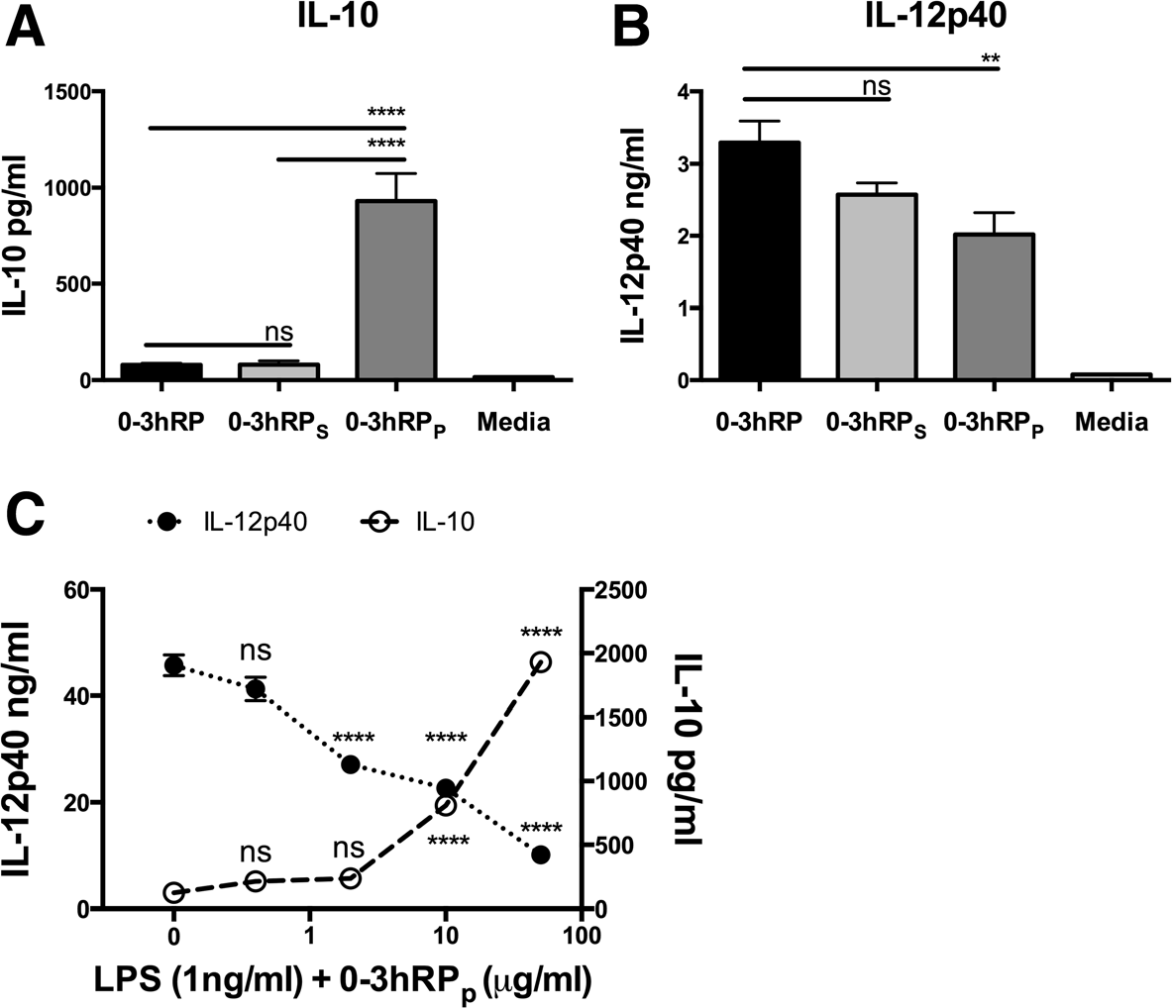
**The pelleted fraction of cercarial E/S products (0-3hRP**_**P**_ **) induces abundant IL-10 from macrophages and blocks LPS driven IL-12p40.** The presence of **(A)** IL-10 and **(B)** IL-12p40 in culture supernatants from BMMΦs exposed to 0-3hRP (50 μg/ml), 0-3hRP_S_ (50 μg/ml), 0-3hRP_P_ (25 μg/ml), or left un-stimulated (Media). **(C)** IL-12p40 (closed circles, left axis) and IL-10 (open circles, right axis) in culture supernatants of BMMΦs exposed to LPS (1 ng/ml) and increasing doses of 0-3hRP_P_. Bars, or symbols, are mean values ± SEM of 6 technical replicates and are representative of three independent experiments. ANOVA and Tukey’s or Dunnett’s test were performed to examine statistically significant differences between selected means (** = p < 0.01; **** = p < 0.0001; ns = p > 0.05).

Macrophages derived from the bone marrow of mice are an abundant and widely used source of naïve cells for *in vitro* studies [13–16]. Consequently, BMMϕs defined on the basis of their expression of CD11b, F4/80, and MHC-II, but not CD11c (Additional file 1: Figure S1) were exposed overnight to 0-3hRP, 0-3hRP_S_, 0-3hRP_P_, or left un-stimulated; 0-3hRP and 0-3hRP_S_ were both used at 50 μg/ml, whilst 0-3hRP_P_ was used at a lower dose (25 μg/ml) due to limited availability of material. Nevertheless, 0-3hRP_P_ induced 10-fold more IL-10 than either 0-3hRP, or 0-3hRP_S_ (Figure 1A, both p < 0.0001). Conversely, IL-12p40 production was significantly lower in BMMϕs exposed to 0-3hRP_P_ compared to 0-3hRP (p < 0.01), although there was no significant difference between IL-12p40 between 0-3hRP and 0-3hRP_S_ (Figure 1B).

As IL-10 induction is significantly increased in response to 0-3hRP_P_, the capacity of this preparation to inhibit IL-12p40 was examined. Therefore, BMMϕs were exposed overnight to LPS (1 ng/ml) in the presence of increasing doses of 0-3hRP_P_. Even low concentrations of 0-3hRP_P_ (2 μg/ml) significantly reduced the amount of IL-12p40 produced by BMMϕs exposed to 1 ng/ml LPS (Figure 1C, p < 0.0001), and although IL-12p40 production was still higher than Media control at the highest dose of 0-3hRP_P_ (50 μg/ml), this was expected, as 0-3hRP_P_ alone is able to induce significant quantities of IL-12p40 (Figure 1B). IL-10 production in the presence of LPS was enhanced in the presence of the greatest concentrations of 0-3hRP_P_ (Figure 1C, p < 0.0001). Notably, IL-12p40 production was significantly impaired at 2 μg/ml of 0-3hRP_P_ (p < 0.0001, Figure 1C), whereas the production IL-10 at this dose was not significantly different compared to BMMϕs stimulated with LPS only (Figure 1C). This observation suggests that inhibition of IL-12p40 is independent of IL-10 as IL-12p40 is significantly reduced even in the absence of IL-10. In line with this hypothesis, increasing doses of unfractionated 0-3hRP were unable to block IL-12p40 production by BMMϕs, stimulated with LPS, despite a significant increase in IL-10 production (p < 0.0001, Additional file 2: Figure S2).

### Sm16 is enriched in the pellet fraction of *S. mansoni* E/S products

**Figure 2:**
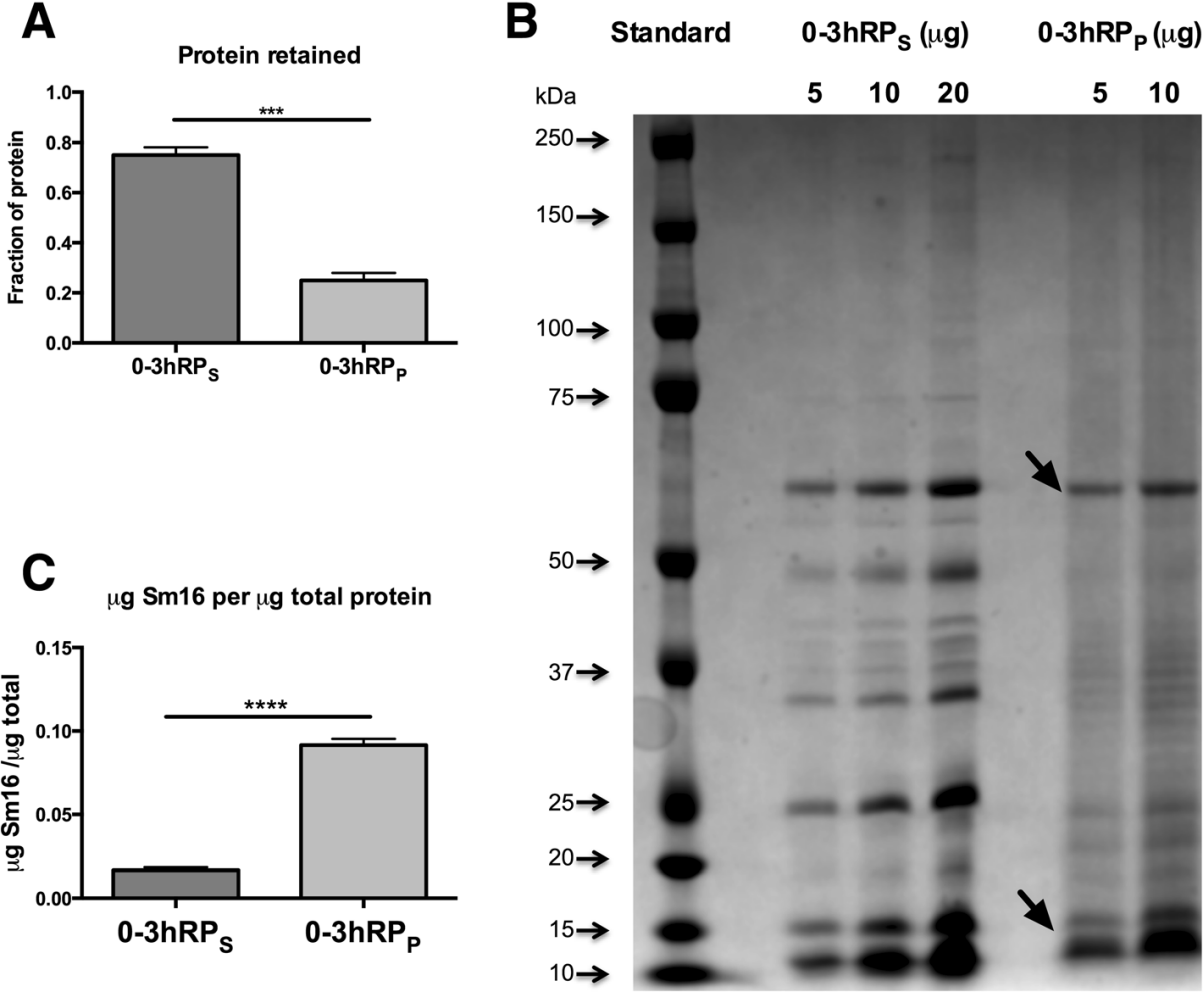
**Sm16 is enriched in pellet fraction of cercarial E/S products.** Three preparations of 0-3hRP were fractionated by ultracentrifugation and the pellet fractions restored to the original volume of each preparation. **(A)** Protein content of each fraction expressed as a percentage of the total protein present in the original preparation. Bars = mean + SEM; statistical significance was tested using two tailed t-test (*** = p < 0.001). **(B)** SDS PAGE gel of 0-3hRP fractions (5, 10 and 20 μg) stained for protein. Black arrows highlight bands identified by mass spectrometry. **(C)** Equivalent volumes of 0-3hRP_S_ (78 μg) and 0-3hRP_P_ (10 μg) based on the original preparation, were processed for Western blot analysis alongside rSm16 (1 μg) probed using rabbit anti-rSm16 antibody, estimated as relative concentration of Sm16 / μg 0-3hRP. Bars are mean + SEM, two tailedt-test show statistically significant differences (**** = p < 0.0001).

As the ability to induce IL-10 in BMMϕs differed between 0-3hRPs and 0-3hRP_P_, the distribution of proteins in the two fractions was assessed by SDS-PAGE. 0-3hRP_S_ retains approximately 75% of the protein content available in the original unfractionated 0-3hRP preparation (Figure 2A), whereas the amount of protein in the 0-3hRP_P_ fraction was much lower (~25%). Analysis by SDS-PAGE revealed a number of discrete bands in the two fractions (Figure 2B) with 0-3hRP_P_ being comprised of a much simpler range than 0-3hRP_S_. Two of the most dominant bands in 0-3hRP_P_ (Figure 2B, black arrows) were identified using MS/MS, with the higher molecular weight band as Invadolysin (M08) (Smp_90100) with a mascot score of 826 and 7 peptides identified, whilst the lower molecular weight band contained Sm16 (Smp_113760), with a mascot score 70 and 2 peptides (Additional file 3: Table S1).

Western blot analysis of equivalent volumes of both fractions, where each fraction was reconstituted to the original starting volume of 0-3hRP used to make the fractions, showed that anti-rSm16 antibody detected the native form of this protein in both 0-3hRP_S_ and 0-3hRP_P_ (Additional file 4: Figure S3), whilst densitometry analysis normalizing to pixel intensity of rSm16, indicates that Sm16 was enriched as a proportion in 0-3hRP_P_ (greater than 5 fold) compared to 0-3hRP_S_ (Figure 2C, p < 0.0001).

### Recombinant Sm16 blocks BMMΦs activation in response to TLR4 and TLR3, but not TLR2 ligands

**Figure 3:**
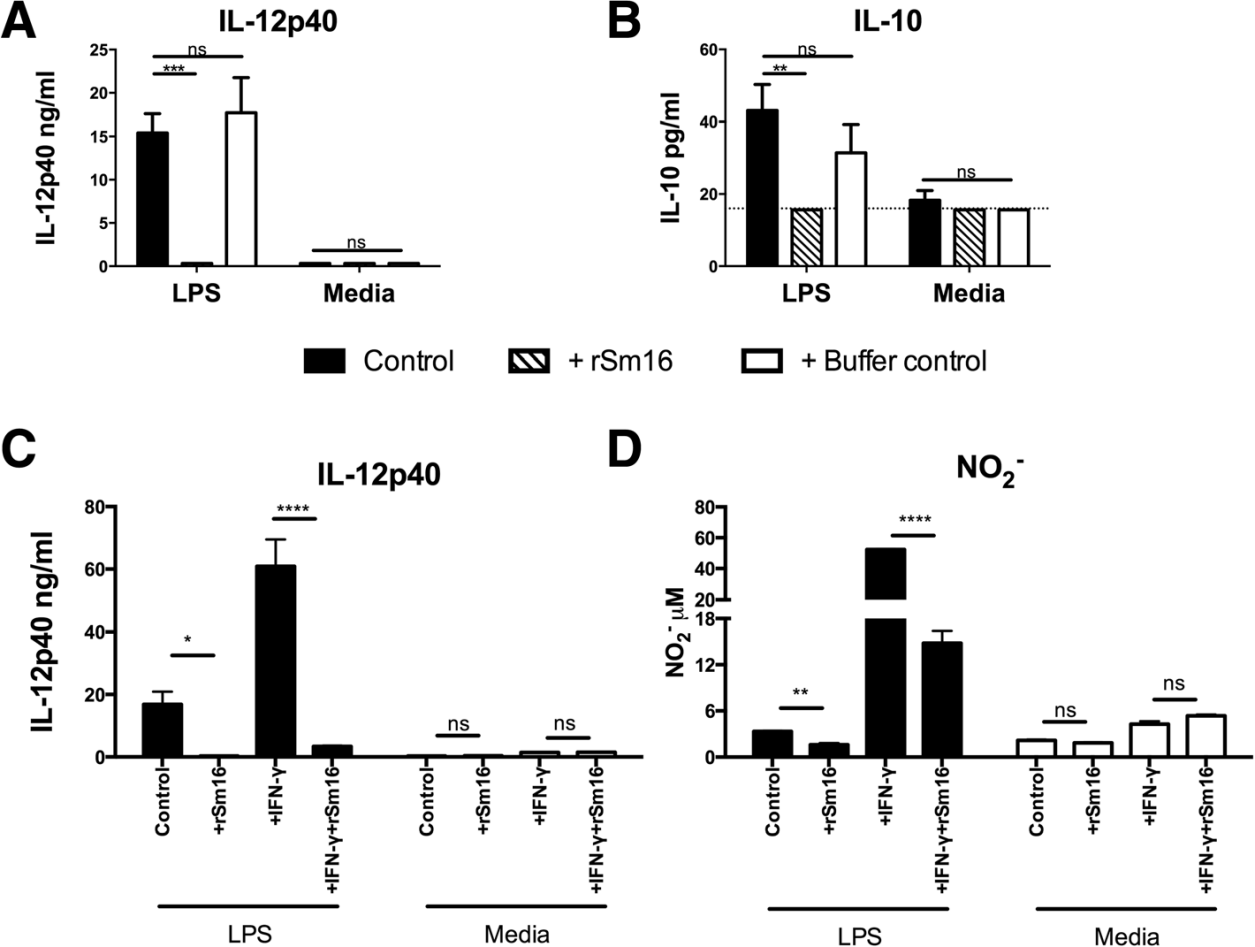
**Recombinant Sm16 blocks activation of BMMΦs in response to LPS and IFN-γ.** The presence of **(A)** IL-12p40 and **(B)** IL-10 in culture supernatants from BMMΦs exposed to LPS (1 ng/ml) (black bars), or Media, in the presence of rSm16 (10 μg/ml) (hatched bars), or an equivalent volume of protein buffer (open bars). **(C)** IL-12p40 and **(D)** nitric oxide (NO_2_^−^) in culture supernatants from BMMΦs exposed to LPS (1 ng/ml) plus IFN-γ (25U/ml) in the presence, or absence of rSm16 (10 μg/ml). Bars = means + SEM of 3 technical replicates. Dotted line represents minimum level of cytokine detection by ELISA. Statistically significant differences tested by ANOVA and Bonferroni’s or Sidak’s test between selected means (* = p < 0.05; ** = p < 0.01; *** = p < 0.001; **** = p < 0.0001; ns = p > 0.05). Results are representative of three independent experiments.

BMMϕs exposed to LPS were unable to produce significant quantities of IL-12p40 and IL-10 when rSm16 (10 μg/ml) was present, whereas the Buffer control (phosphate buffer pH 7.5, containing 0.45 M NaCl to prevent aggregation of rSm16) had no effect (Figure 3A &B). Macrophage function is modulated *in vivo* by several cytokines, particularly IFN-γ, in the presence of ligands for TLR4. In this context, whilst IL-12p40 production to LPS in the presence of IFN-γ (25U/ml) was enhanced, the addition of rSm16 significantly reduced IL-12p40 production (Figure 3C, p < 0.0001). Furthermore, whilst a small amount of NO_2_^−^ was produced by BMMϕs in response to LPS, this was reduced by the presence of rSm16 (Figure 3D, p < 0.01). Activation induced by IFN-γ, greatly enhanced the levels of NO_2_^−^ detected; however again rSm16 significantly reduced the levels of levels of NO_2_^−^ (Figure 3D, p < 0.0001).

**Figure 4:**
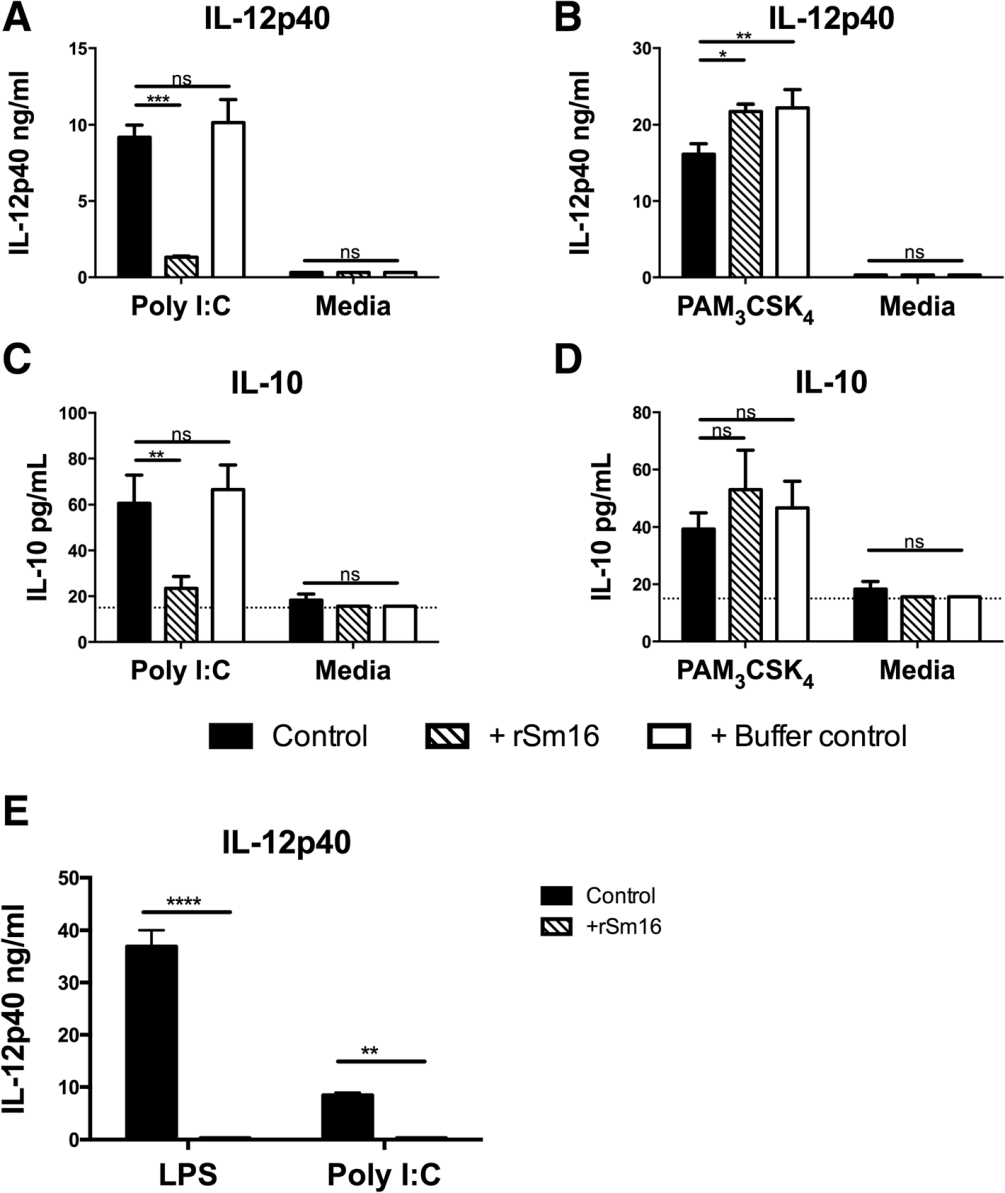
**Recombinant Sm16 blocks cytokine production in BMMΦs exposed to TLR4 and TLR3, but not TLR2 ligands. (A-B)** IL-12p40 and **(C-D)** IL-10 in culture supernatants from BMMΦs exposed to **(A&C)** Poly I:C (25 μg/ml), **(B & D)** Pam3CSK4 (5 μg/ml), or Media, plus rSm16 (10 μg/ml) (hatched bars) or an equivalent volume of protein buffer (open bars). Stimulus only controls (black bars) are also given. **(E)** BMMΦs from TLR2^−/−^ mice exposed to LPS (1 ng/ml), or Poly I:C (25 μg/ml) (solid bars), plus rSm16 (10 μg/ml) (hatched bars), and supernatants tested for IL-12p40 by ELISA. Means + SEM of 3 technical replicates are presented. Dotted line represents minimum level of cytokine detection. ANOVA and Bonferroni’s or Sidak’s test were performed to examine statistically significant differences between the means (* = p < 0.05; ** = p < 0.01; *** = p < 0.001; **** = p < 0.0001; ns = p > 0.05). Results are representative of three independent experiments.

With respect to ligands for other TLRs, BMMϕs stimulated with 25 μg/ml Poly I:C (ligand for TLR3) and rSm16 were also unable to produce IL-12p40 (Figure 4A) or IL-10 (Figure 4C). However, the presence of rSm16 had no effect on cytokine production in BMMϕs exposed to 5 μg/ml Pam3CSK4 (Figure 4B & D). Therefore, whilst rSm16 prevented TLR4 and TLR3 mediated activation of BMMϕs, it appears to be unable to block signalling from TLR2.

Unlike LPS, Pam3CSK4 and Poly I:C, which are recognized by only one TLR, *S. mansoni* cercarial secretions require both TLR2 and TLR4 to induce cytokine production (Sanin *et. al.*, manuscript in preparation). BMMϕs stimulated with 50 μg/ml 0-3hRP alone or treated with rSm16, or buffer control, produced significantly less IL-12p40 (p < 0.01, Additional file 5: Figure S4A) and significantly more IL-10 (p < 0.001, Additional file 5: Figure S4B). Thus, cytokine production by BMMϕs stimulated with 0-3hRP, supplemented with rSm16, is reminiscent of cytokine production by these cells exposed to 0-3hRP_P_ (Figure 1A & B). This is in line with our findings that 0-3hRP_P_ has proportional more Sm16 than the unfractionated antigen. As BMMϕs require TLR2 to respond to 0-3hRP (Sanin *et. al.*, manuscript in preparation), Sm16 too could assert its inhibitory function through this receptor. To confirm this, BMMϕs from TLR2^−/−^ mice were stimulated with LPS, or Poly I:C, in the presence of rSm16. Both ligands induced robust IL-12p40 production in TLR2^−/−^ BMMϕs, but in both cases this was completely ablated by rSm16 (Figure 4E), demonstrating that rSm16 acts on BMMϕs independently of TLR2.

### Recombinant Sm16 is taken up by BMMΦs using a distinct processing pathway

**Figure 5:**
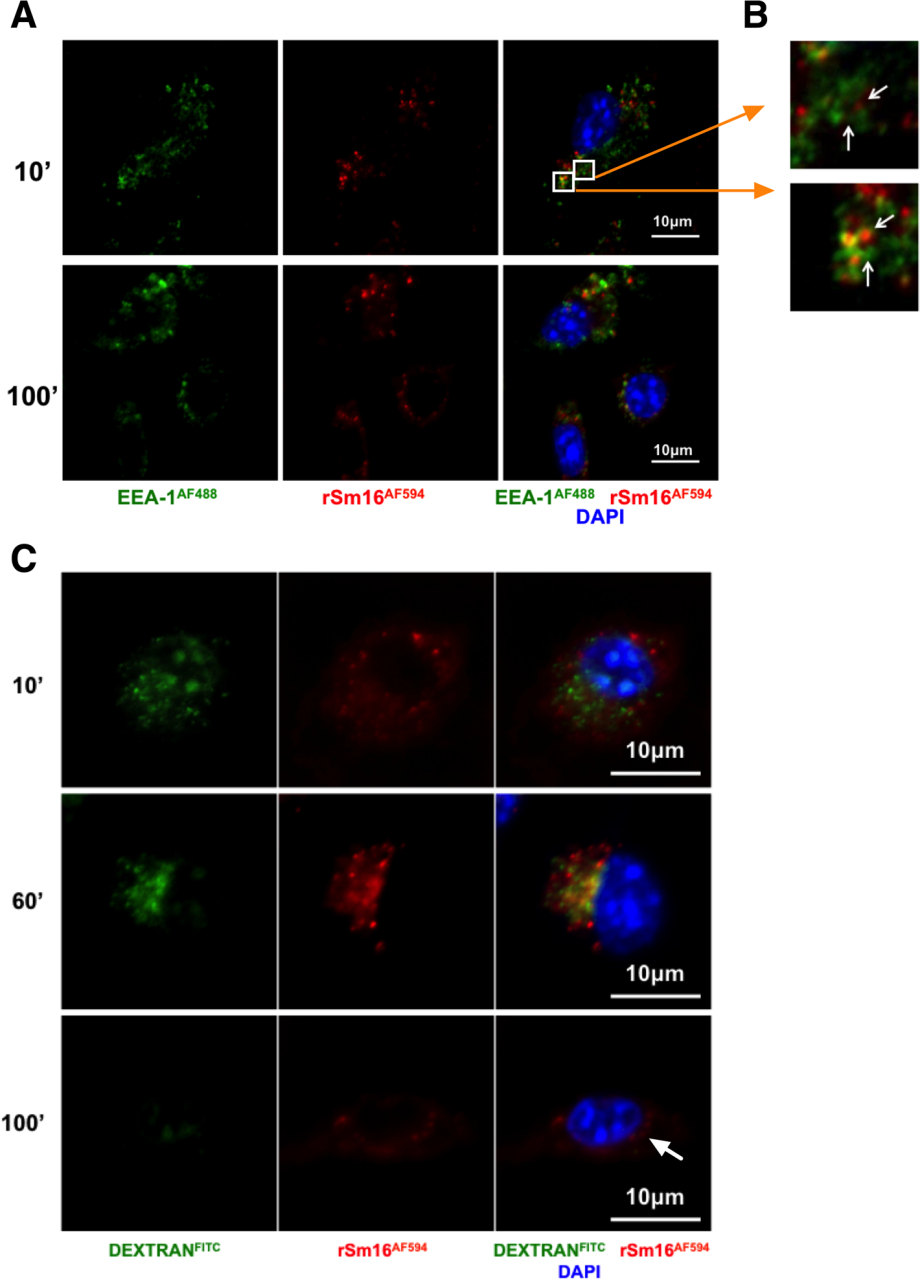
**Uptake of fluorescently labelled rSm16 by BMMΦs.** Representative confocal images of **(A)** BMMΦs exposed to labelled rSm16^AF594^ (red) stained with DAPI (blue) within EEA-1^+^ endosomes (green) 10 min and 100 min after ligand stimulation. **(B)** Insert showing EEA-1^+^ endosomes containing rSm16^AF594^ (2 μm x 2 μm) **(C)** BMMΦs exposed for 100 min to rSm16^AF594^ (red) and DEXTRAN^FITC^ (green) washed and imaged after 10, 60 and 100 min. (63x objective, scale bar = 10 μm; acquired using a Zeiss LSM 710 invert microscope).

BMMΦs exposed to rSm16 labelled with AF594 (rSm16^AF594^), revealed that rSm16 was closely associated with EEA-1 at 10 min, and as late as 100 min after stimulation (Figure 5A). Indeed, EEA-1 appeared to surround rSm16 (Figure 5B, white arrows on inserts), staining which is frequently observed in immunofluorescence microscopy for EEA-1 [17], suggesting that rSm16 persists in early endosomes. This prolonged retention of rSm16 in early endosomes is reminiscent of previous observations for 0-3hRP [3].

The cellular fate of rSm16 in BMMϕs compared to readily processed DEXTRAN, as a marker of material being present in the phagolysosomes [18], demonstrated that 10 and 60 min after removal of these stimuli, both rSm16^AF594^ and DEXTRAN^FITC^ were found in separate and discrete intracellular BMMϕs compartments (Figure 5C). Whilst rSm16^AF594^ was abundant in the periphery of the cell, DEXTRAN^FITC^ was closer to the nucleus. By 100 min, only a faint signal was detected for DEXTRAN^FITC^. The presence of rSm16^AF594^ was also greatly reduced, yet notably foci were still visible in the perinuclear region (Figure 5C, white arrows).

### Recombinant Sm16 treated BMMΦs exhibit delayed antigen processing

**Figure 6:**
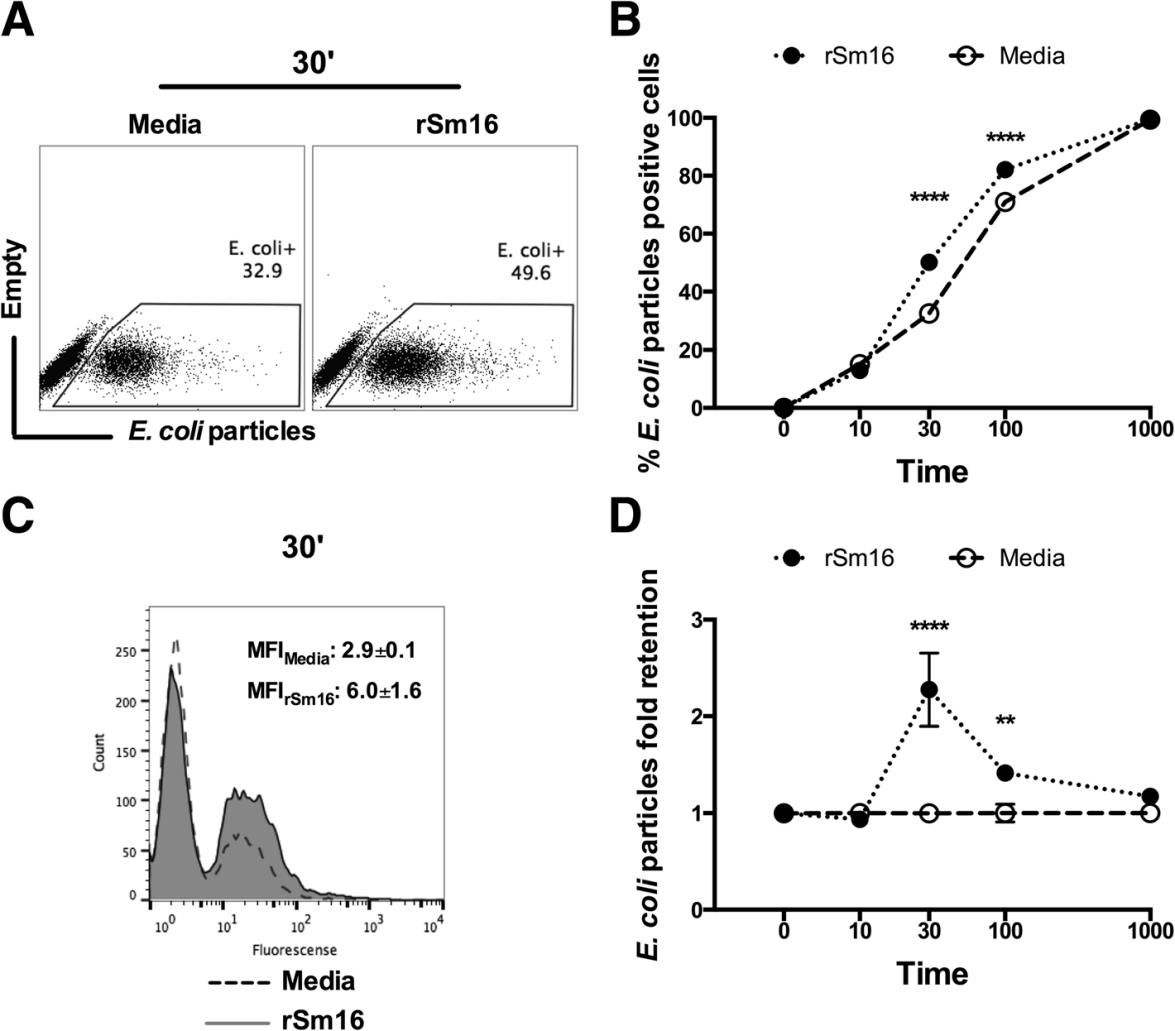
**Recombinant Sm16 delays antigen processing by BMMΦs.** BMMΦs were exposed for 10, 30, 100 or 1000 min to AlexaFluor®488 conjugated *E. coli* BioParticles® (100 particles per cell) in the absence (Media, open circles), or presence of rSm16 (10 μg/ml) (closed circles). **(A)** Representative flow plots of BMMΦs 30 min after exposure to *E. coli* particles. **(B)** Mean percentages ± SEM of BMMΦs containing labelled *E. coli* BioParticles at each time point, and **(C)** representative overlaid histograms including the MFI ± SD of each group. **(D)** Mean ± SEM fold-retention of labelled *E. coli* BioParticles within rSm16-treated BMMφs, relative to the respective MFI of BMMΦs treated with Media control, arbitrarily set to 1. Statistically significant differences between the means of rSm16 treated and Media controls at each time point were examined by ANOVA and Sidak’s test (** = p < 0.01; **** = p < 0.0001).

Prolonged retention of rSm16 in BMMϕs suggested that these cells might have a partial disruption of normal antigen processing. To address this question BMMϕs were treated with rSm16 or left untreated (Media) and subsequently exposed to AlexaFluor®488 conjugated *E. coli* BioParticles® (100 particles per cell) (Figure 6). The percentage of cells containing *E. coli* particles was determined to be significantly higher in rSm16 treated BMMϕs compared to Media control after 30 and 100 min (p < 0.0001, Figure 6A & B) post exposure. The median fluorescence intensity (MFI) of the population (representative overlaid histogram after 30 min given in Figure 6C), as a measure of *E. coli* particles within BMMϕs, was used to calculate the fold increase in the retention of Sm16 antigen, setting Media control arbitrarily to 1 (Figure 6D). In line with increased percentage of positive cells, BMMϕs treated with rSm16 retained 2-fold more *E. coli* particles after 30 min (p < 0.0001) and this retention was still evident (albeit to a lesser degree) after 100 min (Figure 6D; p < 0.01).

## Discussion

In the present study we show that Sm16 is a major component of *S. mansoni* cercarial E/S products and as individuals living in areas endemic for schistosomiasis are liable to be frequently exposed to infective *S. mansoni* cercariae their E/S products, including Sm16, have the potential to provide a major stimulant of the innate immune system. Cercarial E/S products are composed of soluble and particulate fractions, with the latter most likely consisting of protein aggregates, which might accumulate in host tissues to a greater extent than the soluble fraction. As 0-3hRP_P_ is produced as a pellet, its effect on BMMϕs might only occur after proteolytic degradation within these cells. Moreover, increased anti-inflammatory activity observed in the pellet fraction of 0-3hRP may be due to its higher Sm16 content (~10%).

We show that rSm16 inhibits cytokine production to TLR3 and TLR4 ligands in murine macrophages in a manner similar to previous observations using human cells where induction of IL-6 and IL-1RA was impaired [10]. Furthermore, we show that Sm16 also prevents IL-12p40 production by macrophages that could restrict the induction of IL-12-driven Th1 cell responses [19–22]. Indeed, multiple exposures of skin to *S. mansoni* cercariae (and therefore greater quantities of E/S products, including Sm16) leads to lymphocytes in the skin draining lymph nodes becoming hypo-responsive in terms of Th cell associated cytokine production (Prendergast *et. al.*, manuscript submitted) [23]. Infection with Sm16 deficient parasites (obtained through RNA silencing, or genetic manipulation) could ideally prove conclusively the role of this protein in the course of a skin infection, but these tools are not yet available [24].

In addition to its ability to inhibit innate immune cell responses to TLR ligands, Sm16 prevented classical activation of macrophages which ordinarily occurs in the presence of IFN-γ leading to polarized Th1 responses [25]. Classically activated macrophages (CAMϕs) have increased bactericidal capacity as they have an elevated production of NO, and also produce elevated levels of IL-12, further supporting a Th1 environment [26]. However, Sm16 blocked IFN-γ activation of macrophages *in vitro*, both by preventing IL-12p40 production and limiting NO production thereby restricting their ability to become classically activated. Consequently, Sm16 has the potential to impair both innate and adaptive immune responses *in vivo* in naturally infected hosts.

Immune modulation by helminth antigens has been traditionally linked to glycosylation, particularly since helminth E/S products are often heavily glycosylated [27–29]. Several proteins present in 0-3hRP are known to be heavily glycosylated [30], and glycans are partly responsible for the uptake of 0-3hRP [14]. However, Sm16 is not glycosylated [30], and its immunomodulatory function is evident when expressed as a recombinant protein in eukaryotic cells from organisms other than *S. mansoni*, that have an inherently different glycosylation machinery [9,10]. Thus, we conclude that the regulatory function of Sm16 is independent of its glycosylation state.

Earlier reports on the inhibitory potential of Sm16 suggest that this occurs at very early stages of TLR signalling, as it can block both NF-κB activation and IRAK1 degradation [10]. Moreover, since 0-3hRP requires TLR2, TLR4 and MyD88 to induce cytokine production (Sanin *et. al.*, manuscript in preparation) [7] and as its uptake is partly mediated by the mannose receptor [14], several other pattern recognition receptors are likely to be required to mediate the function of 0-3hRP. However, direct competition with TLR ligands as a mechanism for Sm16 inhibition seems unlikely, as TLR ligands have diverse chemical structures. Furthermore, the fact that TLR4 and TLR3 use different adaptor molecules (MyD88 *vs.* TRIF) [31], but are both inhibited by Sm16, suggests that the inhibitory mechanism involving Sm16 is independent of either receptor. Moreover, TLR2 is able to scape the inhibitory effect of Sm16, further suggesting that adaptor molecules and downstream signalling pathways common between the three TLRs are unlikely to be the target of Sm16. Other helminth products inhibit TLR driven cytokine production by sequestration of downstream signalling machinery [32], and 0-3hRP is known to have altered endosomal processing [3]. If Sm16 exerts its function by limiting the availability of TLR signalling machinery, its effect would be then independent of TLR binding. To confirm this conclusion, we demonstrated that TLR2 signalling was indeed not required for the inhibitory action of Sm16. Thus, Sm16 inhibits TLR signalling using a mechanism that does not depend on direct binding to a TLR.

Sm16 was rapidly taken into macrophages and remained in early endosomes for a prolonged time (100 min), reminiscent of 0-3hRP [3] and was not rapidly degraded. On the other hand, DEXTRAN, which is widely used as a lysosome tracker [33,34], was taken up within 10 minutes into a different intracellular compartment and then speedily degraded as it reached the lysosomes. In contrast, the intracellular fate of Sm16 had much slower degradation rates, further confirming our earlier observation of retention in early endosomes. Sm16 was also able to increase the retention of *E. coli* particles in macrophages, suggesting that overall antigen processing in these cells might be altered by Sm16. Phagosome maturation is influenced by TLR4 signalling [35], which Sm16 is able to block. Thus, the observed delayed antigen processing evidenced in this report could be as a result of Sm16 mediated TLR inhibition. However, as ligation of TLR4 can direct signalling from phagosomes [36,37], this opens up the possibility that Sm16 might be blocking TLR signalling by limiting antigen trafficking to these organelles.

## Conclusions

We conclude that the particulate/pellet fraction of *S. mansoni* cercarial E/S material contains a significantly greater proportion of Sm16 than the soluble fraction, which may explain why the pellet fraction has a greater propensity to induce regulatory IL-10. The inhibitory activity of Sm16 operates upon TLR4 and TLR3 induced signalling but is not mediated by direct interaction with TLR2. Moreover, Sm16 is able to prevent classical activation of macrophages in the context of IFN-γ stimulation. The mechanism employed by Sm16 to inhibit macrophage activation is likely to be linked with the rapid uptake and retention of this protein, which has a membrane binding properties [9], leading it to be quickly internalized. However, the rapid uptake of Sm16 does not lead to fast degradation, or indeed lysosomal trafficking, but instead results in retention in early endosomes leading to delayed processing. As with other helminth products, retention within intracellular components may possibly be responsible for mediating sequestration of essential TLR signalling machinery that subsequently blocks stimulation of specific TLRs [32]. Moreover, it appears that Sm16 extends this processing “defect” to *E. coli* particles, which exhibit enhanced retention in macrophages exposed to this protein, which could suggest that Sm16 prevents TLR signalling from within phagosomes. This could help explain why repeated exposure to *S. mansoni* cercariae, consequently leading to greater exposure to particulate fraction of cercarial secretions containing Sm16, leads to the inhibition of APC function and T cell responses in the course of infection [23].

## Additional files

Additional file 1: Figure S1. Characterization of bone marrow cells as macrophages. Bone marrow cells cultured for 7 days were stained with (**A**) a viability dye and (**B**) labelled with antibodies against F4/80, CD11b, MHC-II and CD11c or relevant antibody isotype controls. Representative flow plots with percentages for numbers of cells within each gate from 7 independent experiments. Additional file 2: Figure S2. Unfractionated cercarial E/S products (0-3hRP) do not block LPS driven production of IL-12p40. The presence of IL-12p40 (closed circles, left axis) and IL-10 (open circles, right axis) in culture supernatants of BMMΦs exposed to LPS (1ng/ml) and increasing doses of 0-3hRP. Symbols are mean values ±SEM of 3 technical replicates and are representative of two independent experiments. ANOVA and Dunnett’s test were performed to examine statistically significant differences between mean of LPS only control and LPS+0-3hRP treated BMMΦs at each dose (**** = p<0.0001; ns = p>0.05). Additional file 3: Table S1. ᅟ Additional file 4: Figure S3. Western blot for Sm16 in fractions of cercarial E/S products. Equivalent volumes of 0-3hRP_S_ (78 μg) and 0-3hRP_P_ (10μg) based on the original preparation, plus an extra lane with 2x 0-3hRP_P_ (20μg), were processed for Western blot analysis alongside rSm16 (1μg) and probed using rabbit anti-rSm16 antibody. Additional file 5: Figure S4. BMMΦs stimulated with recombinant Sm16 and *S. mansoni* cercarial secretions (0-3hRP) produced enhanced IL-10 and reduced IL-12p40. The presence of (**A**) IL-12p40 and (**B**) IL-10 in culture supernatants from BMMΦs exposed to 0-3hRP (50μg/ml) (black bars), or Media, in the presence of rSm16 (10μg/ml) (hatched bars), or an equivalent volume of protein buffer (open bars). Bars are means +SEM of 3 technical replicates. Dotted line represents minimum level of cytokine detection. Statistically significant differences tested by ANOVA and Sidak’s test between selected means (** = p<0.01; *** = p<0.001; **** = p<0.0001; ns = p>0.05). Results are representative of three independent experiments.
