# Supplementary figures and images for: Sm16, a major component of Schistosoma mansoni cercarial excretory/secretory products, prevents macrophage classical activation and delays antigen processing
Source: Parasit Vectors. 2015 Jan 6;8:1. doi: 10.1186/s13071-014-0608-1 (PMC4297449; doi:10.1186/s13071-014-0608-1)

Figure S1. Characterization of bone marrow cells as macrophages

A

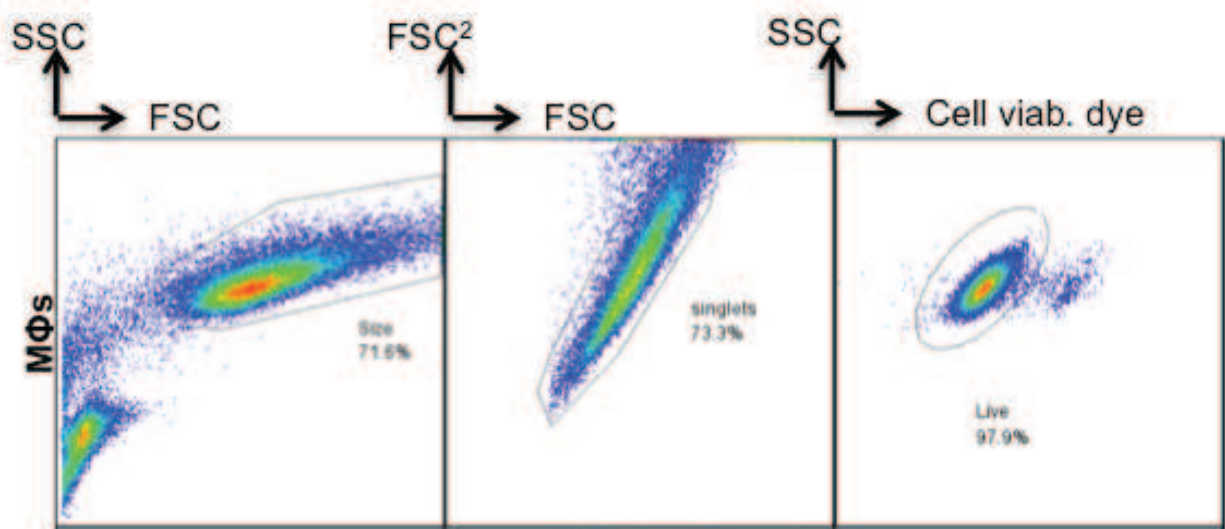

B

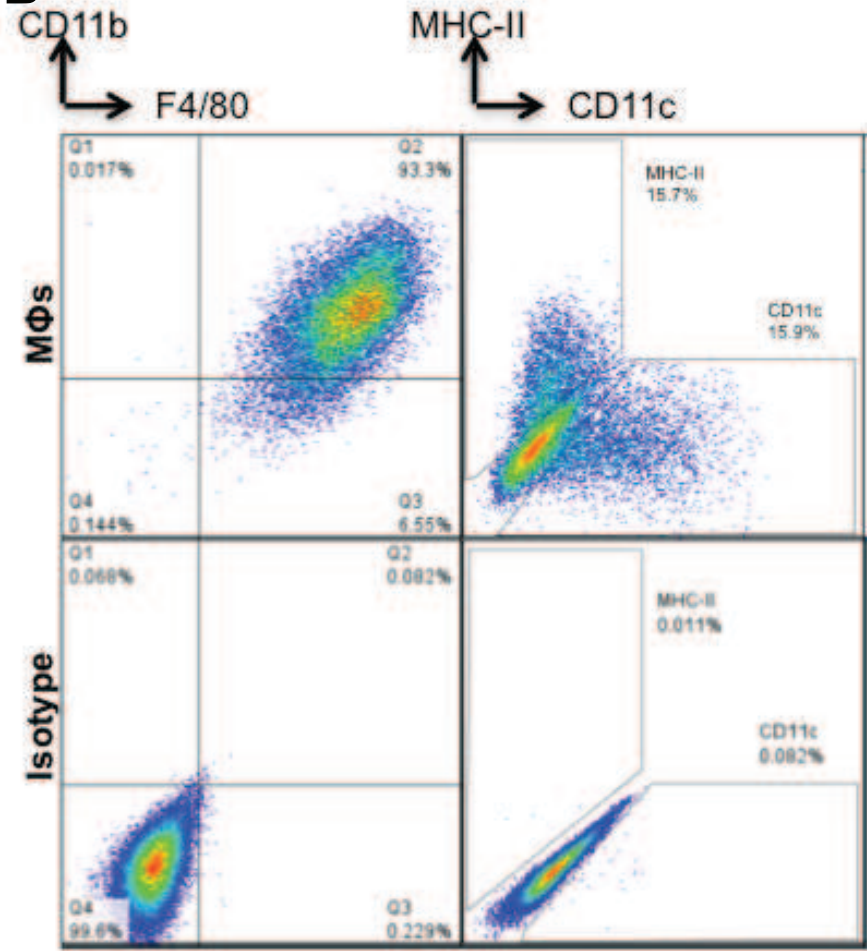

Supplement: Additional file 1: Figure S1. — Characterization of bone marrow cells as macrophages. Bone marrow cells cultured for 7 days were stained with (A) a viability dye and (B) labelled with antibodies against F4/80, CD11b, MHC-II and CD11c or relevant antibody isotype controls. Representative flow plots with percentages for numbers of cells within each gate from 7 independent experiments. [file 13071_2014_608_MOESM1_ESM.pdf]

**Figure S2. Unfractionated cercarial E/S products (0-3hRP) are unable to block LPS driven IL-12p40.**

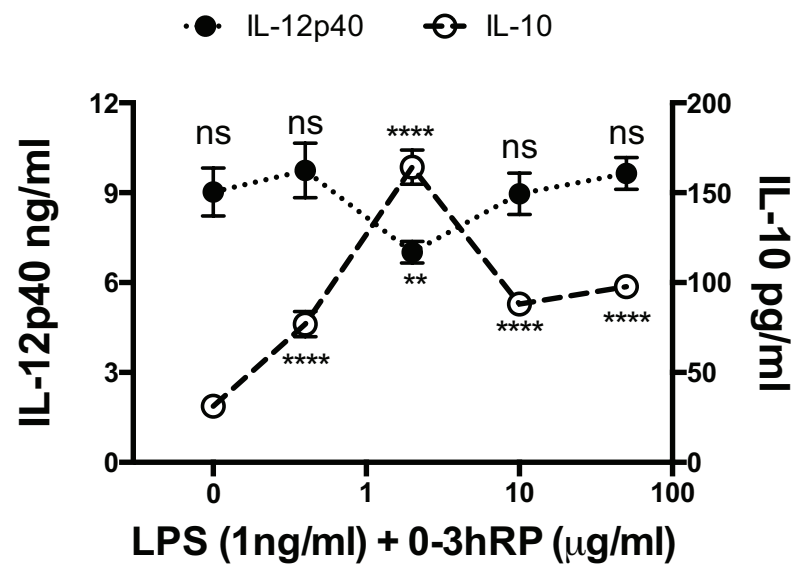

Supplement: Additional file 2: Figure S2. — Unfractionated cercarial E/S products (0-3hRP) do not block LPS driven production of IL-12p40. The presence of IL-12p40 (closed circles, left axis) and IL-10 (open circles, right axis) in culture supernatants of BMMΦs exposed to LPS (1ng/ml) and increasing doses of 0-3hRP. Symbols are mean values ±SEM of 3 technical replicates and are representative of two independent experiments. ANOVA and Dunnett’s test were performed to examine statistically significant differences between mean of LPS only control and LPS+0-3hRP treated BMMΦs at each dose (**** = p<0.0001; ns = p>0.05). [file 13071_2014_608_MOESM2_ESM.pdf]

**Figure S3. Western blot for Sm16 in fractions of cercarial E/S products**

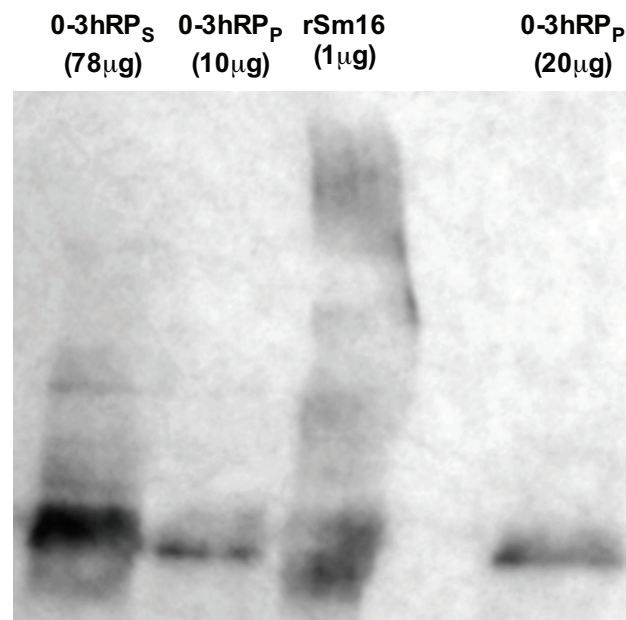

Supplement: Additional file 4: Figure S3. — Western blot for Sm16 in fractions of cercarial E/S products. Equivalent volumes of 0-3hRPS (78 μg) and 0-3hRPP (10μg) based on the original preparation, plus an extra lane with 2x 0-3hRPP (20μg), were processed for Western blot analysis alongside rSm16 (1μg) and probed using rabbit anti-rSm16 antibody. [file 13071_2014_608_MOESM4_ESM.pdf]
