# Supplementary material for: Sm16, a major component of Schistosoma mansoni cercarial excretory/secretory products, prevents macrophage classical activation and delays antigen processing
Source: Parasit Vectors. 2015 Jan 6;8:1. doi: 10.1186/s13071-014-0608-1 (PMC4297449; doi:10.1186/s13071-014-0608-1)
Supplement: Additional file 3: Table S1. — ᅟ [file 13071_2014_608_MOESM3_ESM.pdf]

Table S1

Identified protein bands					
**Name**	**Gene ID**	**Mascot score**	**Peptide Score**	**Peptides identified**	**Peptide mass (Da)**
Invadolysin	Smp_090100	826	30	K.ALNFWER.T	935.4714
			51	K.AFGICAVGR.F	950.4852
			35	R.CPLIQPMR.S	1014.515
			83	R.GCVEPVFYTDGR.T	1399.615
			94	K.QIQGEIICPNPNIACR.N	1882.908
			102	K.TMLIGTAIHELGHALGFVK.S	2008.077
			65	K.NLGCSFVMESCYAYMMR.M	2118.828
			137	R.LPPTEQYFNDPTQGGTSVLNDR.C	2449.125
			79	R.SFFNEPLVTYCDHQLNIPVAQR.G	2648.251
			151	K.ALCYDHPVPDQYASGCAMGGGGGDIR.D	2724.123
Sm16	Smp_113760	70	31	K.GRHHIYK.V	910.4909
			41	K.SICNEIKR.S	1019.542
