# Supplementary material for: Sm16, a major component of Schistosoma mansoni cercarial excretory/secretory products, prevents macrophage classical activation and delays antigen processing
Source: Parasit Vectors. 2015 Jan 6;8:1. doi: 10.1186/s13071-014-0608-1 (PMC4297449; doi:10.1186/s13071-014-0608-1)

Figure S4. BMMφs stimulated with recombinant Sm16 and *S. mansoni* cercarial secretions (0-3hRP) produced enhanced IL-10 and reduced IL-12p40.

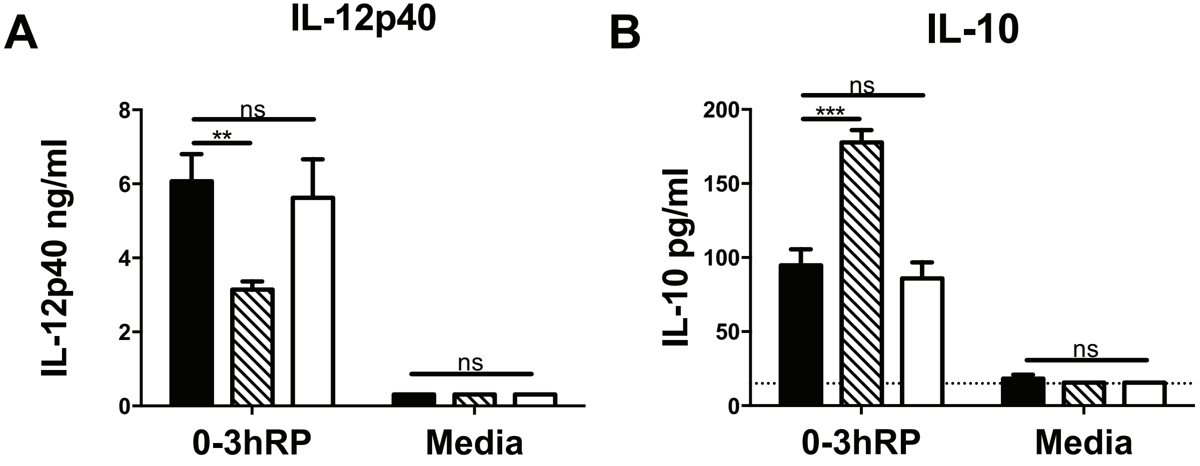

Supplement: Additional file 5: Figure S4. — BMMΦs stimulated with recombinant Sm16 and S. mansoni cercarial secretions (0-3hRP) produced enhanced IL-10 and reduced IL-12p40. The presence of (A) IL-12p40 and (B) IL-10 in culture supernatants from BMMΦs exposed to 0-3hRP (50μg/ml) (black bars), or Media, in the presence of rSm16 (10μg/ml) (hatched bars), or an equivalent volume of protein buffer (open bars). Bars are means +SEM of 3 technical replicates. Dotted line represents minimum level of cytokine detection. Statistically significant differences tested by ANOVA and Sidak’s test between selected means (** = p<0.01; *** = p<0.001; **** = p<0.0001; ns = p>0.05). Results are representative of three independent experiments. [file 13071_2014_608_MOESM5_ESM.pdf]
